# Excess demand prediction for bike sharing systems

**DOI:** 10.1371/journal.pone.0252894

**Published:** 2021-06-17

**Authors:** Xin Liu, Konstantinos Pelechrinis

**Affiliations:** Department of Informatics and Networked Systems, School of Computing and Information, University of Pittsburgh, Pittsburgh, PA, United States of America; Univ. Lyon, ENTPE, Univ. Gustave Eiffel, FRANCE

## Abstract

One of the most crucial elements for the long-term success of shared transportation systems (bikes, cars etc.) is their ubiquitous availability. To achieve this, and avoid having stations with no available vehicle, service operators rely on *rebalancing*. While different operators have different approaches to this functionality, overall it requires a demand-supply analysis of the various stations. While trip data can be used for this task, the existing methods in the literature only capture the *observed* demand and supply rates. However, the *excess* demand rates (e.g., how many customers attempted to rent a bike from an empty station) are not recorded in these data, but they are important for the in-depth understanding of the systems’ demand patterns that ultimately can inform operations like rebalancing. In this work we propose a method to estimate the excess demand and supply rates from trip and station availability data. Key to our approach is identifying what we term as excess demand pulse (EDP) in availability data as a signal for the existence of excess demand. We then proceed to build a Skellam regression model that is able to predict the difference between the total demand and supply at a given station during a specific time period. Our experiments with real data further validate the accuracy of our proposed method.

## Introduction

During the past few years, urban and transportation planners have come to realize that if we want our cities to thrive and lead the way to a sustainable future, a turn to multimodal and shared transportation is needed. This has led to the fast growth of shared transportation options, with shared bike systems enjoying particularly wide adoption across the world [1]. The first generation of shared bike systems involves docks/stations where a commuter can rent a bike from and return a bike to after a ride. The second wave of shared bike systems includes dockless infrastructure that allows bikes to self-lock and hence, to be returned to (and picked up from) any place in a city. However, despite the seemingly convenience dockless systems offer, only 4% of all the trips in 2017 were made from dockless bikes [2]. While this number might have increased during the last few years, cities are still reluctant for their wide adoption due to a variety of reasons (e.g., littering of sidewalks, parks and other public spaces) [3].

For docked shared bike systems, one of the largest operational expenses is associated with *rebalancing* [4]. Rebalancing aims at redistributing bikes from stations with excess supply to stations with excess demand in order to assure (ubiquitous) availability for customers. The latter is very important for customer satisfaction and retention, and of course, for the survival of the systems themselves and their associated societal and environmental benefits. Typically the operator utilizes one or more trucks to transfer bicycles from stations with high supply and low demand (e.g., full stations) to ones with low supply and high demand (e.g., empty stations). This happens usually in a reactive fashion, i.e., availability levels are monitored across the stations and once it drops below a threshold rebalancing is triggered. Nevertheless, there is a proactive element as well, since operators analyze historical trip data to identify the demand and supply of each station for different days and times. However, simply counting trips from and to a station paints only part of the demand/supply rates, since zero trips from a station, do not necessarily mean there is no demand for bikes. If there are no bikes available, any customer attempting to make a trip will not be able to rent a bike, thus, contributing to the excess demand of the system. This information is not captured when looking purely at the number of trips [5–10]. Similarly, when it comes to supply of bikes, a full station will result in bikes not being able to be returned to this station [11–14]. However, this does not mean that the supply rate is zero.

In this study we provide an empirical approach for estimating the ***excess demand and supply*** levels in a shared transportation system borrowing ideas from the parking literature—and in particular research on estimating the percentage of traffic looking for a parking spot [15]. As we will elaborate in Section “Excess demand estimation”, this estimation requires additional data from trip-logs, namely, the number of bikes and docks available at the bike station. At a high-level, for computing the excess demand (similarly for the excess supply, i.e., a station is full so a customer cannot return her currently rented bike), we focus on periods with 0 available bikes (full stations respectively). Then if a bike is returned at time *t*_1_ and the first rental happens at time *t*_2_, this time interval [*t*_1_, *t*_2_] is very important for estimating the bike excess demand, which is part of the total bike demand (i.e., the trips observed from the data, and the trips that were not possible to be completed due to lack of bikes). A similar approach can be used for the supply side, i.e., for the cases where a user wants to return a bike but the station is full and hence, she cannot store it at the dock.

Consequently, we show that even though the total demand (or total supply) can be predicted at an acceptable level through a Poisson regression, the correlations between the supply and demand side—each of which modeled through an independent Poisson distribution—are high enough that leads to biased results when using them for predicting the net total demand (i.e., the difference between the total demand and supply). The net total demand is our target of prediction since it provides direct insights for the bike operator to decide the number of bikes to be rebalanced. To overcome this challenge we further develop a Skellam regression model that directly models the net total demand in a station, and, thus, accounting for this correlation.

In summary, the contributions of our study are twofold:

- We introduce an approach for estimating the excess demand of bike sharing systems using high-level ideas from queuing theory. Key to our approach is identifying temporal segments—which we term excess demand pulse (EDP)—in the bike availability data, that include changes in the availability from zero (i.e., no bikes at the dock) to non-zero (i.e., someone returned a bike). After introducing the theoretical underpinnings of our method, we verify through simulations its ability to estimate the excess demand present in the system. We consequently apply our approach on data obtained from a real bike sharing system, Chicago’s Divvy, to estimate the excess demand present in the system.
- Using the estimated excess demand, we learn a Skellam regression model through maximum likelihood estimation for predicting the net total demand, which shows advantages over other alternative models, both in terms of predictive performance, as well as, interpretability. Moreover, our Skellam regression model, as a generalized linear model, allows us to get a better estimation of the uncertainty of our prediction, since we essentially obtain the whole probability distribution of our dependent variable.

### Related literature

There have been several studies on demand prediction in bike sharing systems, i.e., the expected number of bikes to be rented and returned at each station. Most of them only consider the observed demand, i.e., the demand reflected in the trip data logged by the system [5–10]. However, the total demand includes also trips that were never realized due to empty docks. To reiterate, we refer to this part of the total demand as excess demand. Failing to involve the excess demand will essentially provide a model that only captures the observed demand of the system, essentially treating any period with zero observed rentals (or returns respectively) as periods of zero demand, which is not true in general.

In a slightly different, but relevant, problem formulation some studies focus on bike availability prediction, i.e, the expected number of bikes available for rental at a station [6, 7, 16–19]. A variety of specifications have been used for the prediction models, including auto-regressive moving average, K Nearest Neighbors, random forest, gradient boosted tree, and neural networks. Hierarchical predictions [9, 20] have also been developed, where stations are firstly clustered into relevant groups (e.g., geographically close) and then, predictions happen at the cluster level.

Some of these studies, such as the one from Schlote *et al*. [16] point out that a popular station may run out of bike quickly if the demand is so high, while others [8] identify “over-demand” stations as those that are full or empty for more than 10 minutes. Then they propose algorithms to classify a station as an “over-demand” one. However, none of these studies attempts to estimate the volume of excess demand.

However, there are studies that attempt to estimate the volume of excess demand using a simple method based on the duration for a station being empty [21–23]. These methods assume that excess demand exists every time there are zero bikes available for rental. They further consider this excess demand to be equal to the observed demand in adjacent time periods. It should be evident that neither of these assumptions are very realistic. A station can be empty and no user is interested in renting a bike from that station, while the excess demand does not have to be equal to the observed demand in adjacent times.

While several distributions have been used to model the bike arrivals (and departures) within a bike sharing, including negative binomial [24], Weibull [25–27] and Poisson [28–33], the latter is the most common choice for this task. Gast *et al*. [19] show through a Kolmogorov-Smirnov test [34] that the trips in the Paris bike sharing system follow a Poisson distribution. In the following Section we use a similar approach to show that the trips in our dataset fit a Poisson distribution as well.

## Materials and methods

### Excess demand estimation

As aforementioned, excess demand is not captured in the recorded consumption of a product, since it appears when there is zero supply. Hence, it is very challenging to estimate it. In this section, borrowing ideas from queuing theory, we will introduce a way to estimate the excess demand. We further simulate the bike rental and return process to show the ability of the proposed approach to estimate the excess demand in a bike sharing system. Then, we apply our approach on data obtained from a real bike sharing system, Chicago’s Divvy, to estimate the excess demand present in the system. Notations used in describing our approaches and models through the paper are shown in Table 1.

**Table 1:** A list of notations used through the paper.

Symbol	Description
*μ*	actual bike departure rate by total demand
$\hat{\mu }$	estimated bike departure rate by total demand
*μ*_*e*_	actual bike departure rate by excess demand
${\hat{\mu }}_{e}$	estimated bike departure rate by excess demand
λ	actual bike arrival rate by total demand
$\hat{\lambda }$	estimated bike arrival rate by total demand
λ_*e*_	actual bike arrival rate by excess demand
${\hat{\lambda }}_{e}$	estimated bike arrival rate by excess demand
*a*	number of available bikes
*τ*_*f*_	EDP length
*τ*_*m*_	the average of multiple *τ*_*f*_
*τ*_*s*_	the average of inter-supply intervals
*t*_*end*_	the end time stamp of the availability curve
*t*_*a*_, *t*_*b*_, *t*_*c*_, *t*_*d*_, *t*_1_, *t*_2_, *t*_3_, *t*_4_	specific time stamps of the availability curve
*N*_*μ*_	total bike demand volume
*N*_λ_	total dock demand volume
*Z*	net total demand volume
$N_{\mu_{o}}$	observed bike demand volume
$N_{\mu_{e}}$	excess bike demand volume
$N_{\lambda_{o}}$	observed dock demand volume
$N_{\lambda_{e}}$	excess dock demand volume
$l_{\mu_{e}}$	duration length for bike excess demand in a 30-minute interval
$l_{\lambda_{e}}$	duration length for dock excess demand in a 30-minute interval

**Fig 1:**
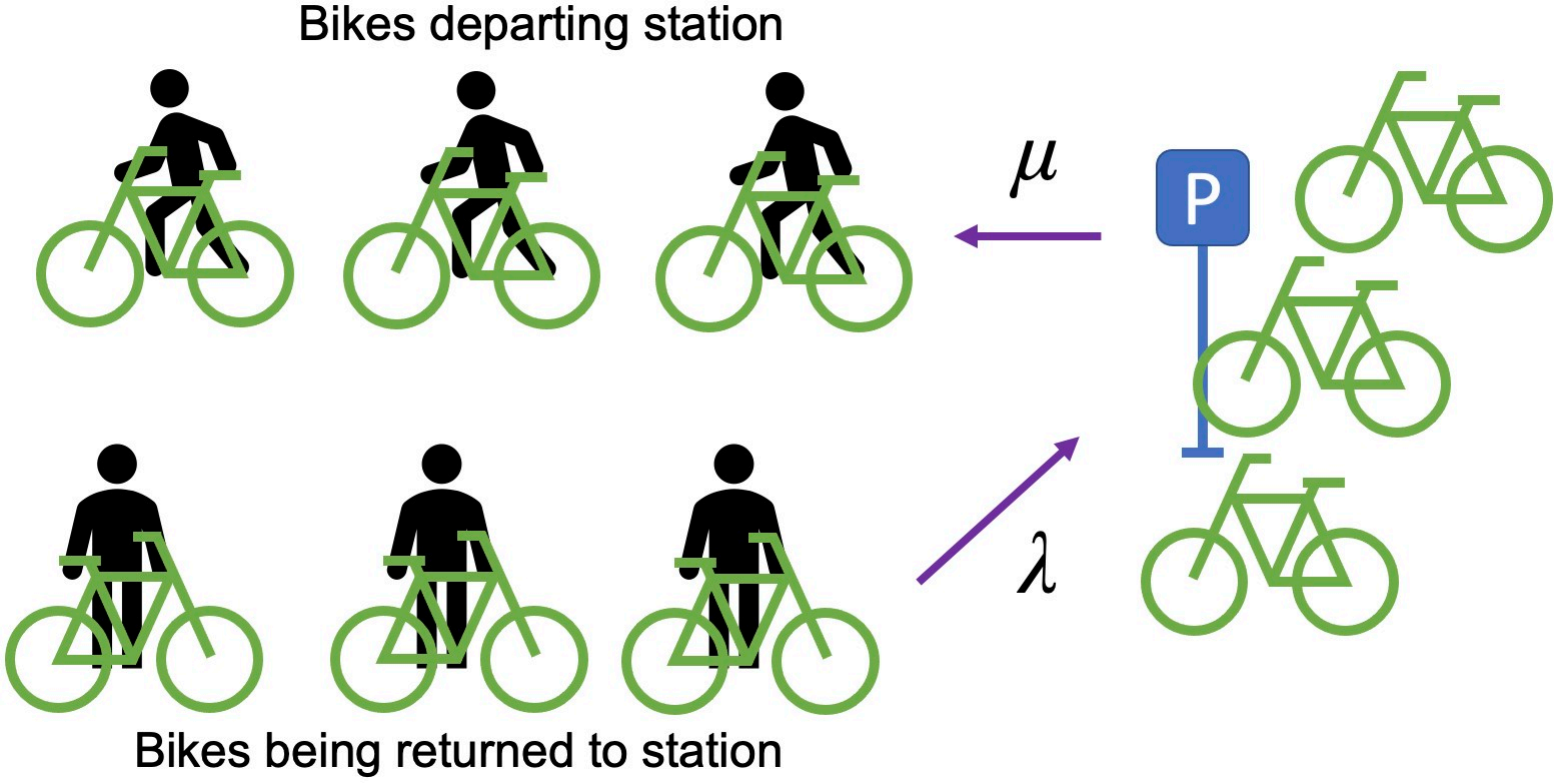
Bike departure and arrival event flows at a bike station.

At a bike station, we generally have two types of event flows occurring as illustrated in Fig 1. One flow represents the bike departure (rental) events, with the number of departures per time unit following a Poisson distribution with intensity *μ* [19]. This also means that the inter-departure time intervals follow exponential distribution with an average of $\frac{1}{\mu }$. The other flow represents the bike arrival (return) events, with the number of arrivals per time unit following Poisson distribution with intensity λ (and similarly the inter-arrival time intervals follow an exponential distribution with average $\frac{1}{\lambda }$). Under the assumption of the flows being independent, we can consider their union as a single flow with mixed types of events [35]. In this mixed flow, the number of events per time unit follows a Poisson distribution with intensity (λ + *μ*), while the inter-event time intervals follow an exponential distribution with average $\frac{1}{\lambda +\mu }$.

**Fig 2:**
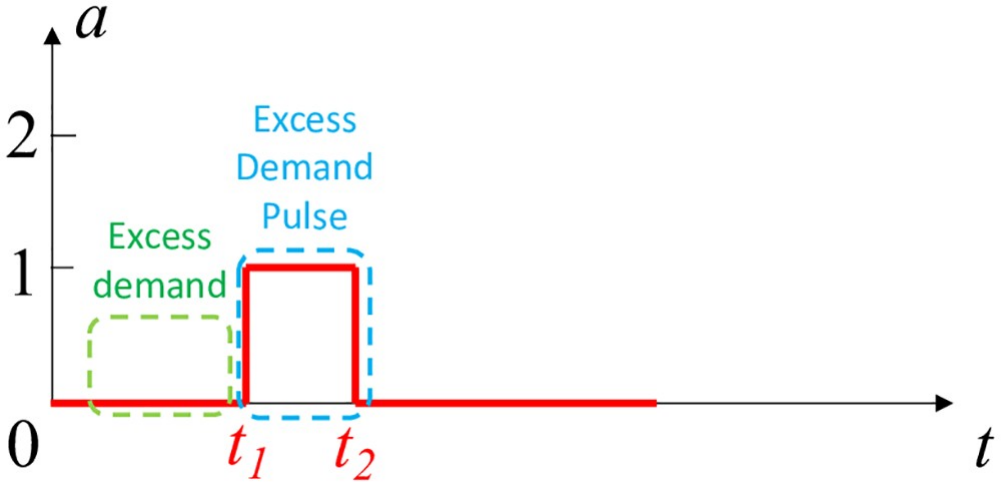
This bike availability curve indicates possible excess demand for *t* ∈ (0, *t*_1_).

**Fig 3:**
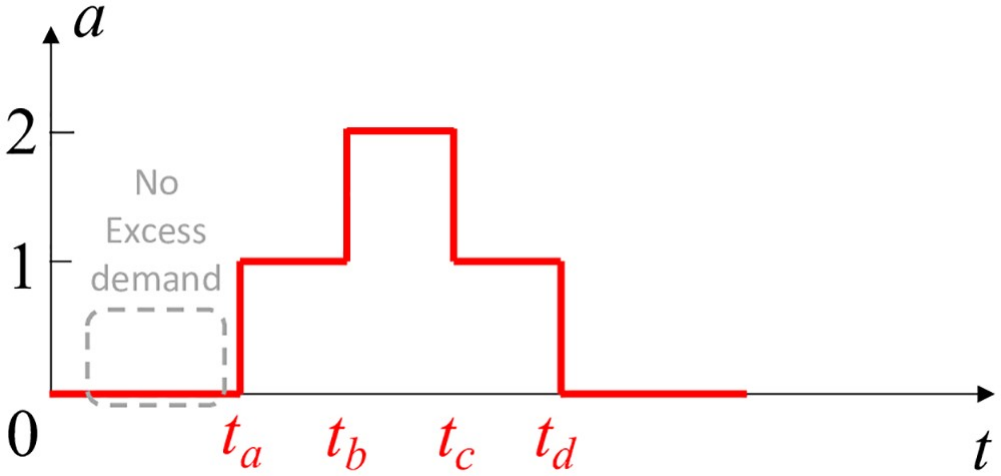
This bike availability curve indicates no excess demand for *t* ∈ (0, *t*_*a*_).

Let us assume that the number of available bikes at a station is *a*. Fig 2 shows a segment of the bike availability curve, where *a* changes from 0 to 1 after a bike arrival at *t*_1_, and goes back to 0 after a rental at *t*_2_. This pattern is central to our estimation of bike excess demand rate (denoted with *μ*_*e*_), and we refer to this curve pattern as excess demand pulse (EDP). We also define *τ*_*f*_ = *t*_2_ − *t*_1_ as EDP length. During the interval (0, *t*_1_), the bike availability is constantly 0, which can be interpreted by someone that there are no events (rentals or returns) happening during that time. However, this is not necessarily true. This constant 0 availability can indeed be due to no events happening during this interval, or due to failed bike rentals, that is, a customer tried to rent a bike but none was available. The pattern captured by the EDP serves as an important signal for the possible presence of excess demand and its degree. Intuitively, the presence of significant excess demand leads to situations where any supply that becomes available is consumed shortly thereafter. At the situation visualized in Fig 2 when the single bike arrives at *t*_1_, it is quickly consumed (rented) at time *t*_2_. In contrast, if we consider the scenario presented in Fig 3, a bike arrives at *t*_*a*_ but it is not consumed *quickly*. Instead, another bike arrives at *t*_*b*_ before a rental. Therefore, any bike demand in this case can be captured well from rental logs, and it is not excess. In other words, the pattern in Fig 3 does not provide evidence for the existence of excess demand.

**Fig 4:**
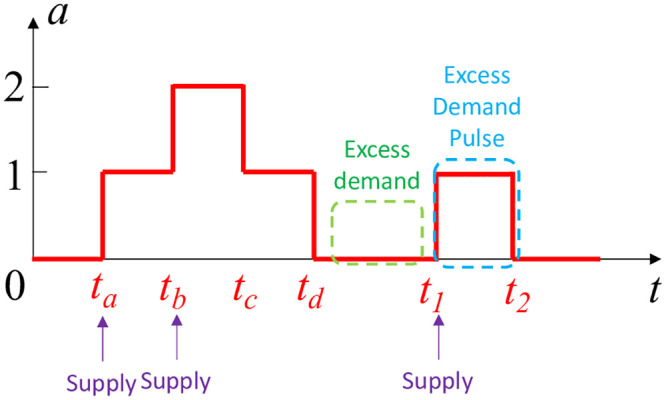
A segment of bike availability curve to illustrate the estimation of excess demand.

Using these observations let us see how we can estimate *μ*_*e*_ through the bike availability curves. Fig 4 depicts a segment of the bike availability curve. Recall that the mixture of arrival and departure flows follows a Poisson distribution with intensity (λ + *μ*). That is, the inter-event intervals of this mixture follow exponential distribution with intensity $\frac{1}{\lambda +\mu }$. If we observe an arrival event followed by departure event, such observation is caused by mixing arrival and departure flows. Thus, in such observation, the interval from the arrival to the departure event follows an exponential distribution with intensity $\frac{1}{\lambda +\mu }$. Thus, *τ*_*f*_ = *t*_2_ − *t*_1_ is a sample from an exponential distribution with average $\frac{1}{\lambda +\mu }$. During a large observation period we will observe *τ*_*F*_ from multiple EDPs, denoting their average value as *τ*_*m*_. By expectation, we should get $\tau_{m}\approx \frac{1}{\lambda +\mu }$. That is, the estimated intensity of the mixed flow is $\hat{\lambda }+\hat{\mu }=\frac{1}{\tau_{m}}$.

We can also calculate the estimated arrival rate $\hat{\lambda }$ from the data. In this paper, we focus on bike sharing systems with docks, so while there is a possibility for *excess supply* in a bike station—e.g., a user tries to return a bike to a full dock—this is not an issue in the presence of bike excess demand. In general, there cannot be bike excess demand and excess supply at the same station during the same time. Therefore, each bike supply (i.e., bike arrival) event is successfully reflected in the bike availability curve when there is bike excess demand present. To reiterate, the inter-supply (i.e., inter-arrival) intervals themselves follow exponential distribution with intensity $\frac{1}{\lambda }$. By obtaining all inter-arrival intervals from the data we can estimate their average denoted as *τ*_*s*_. For example, in the segment in Fig 4, we have arrivals at *t*_*a*_, *t*_*b*_, *t*_1_, resulting in $\tau_{s}=\frac{\left(t_{b}-t_{a}\right)+\left(t_{1}-t_{b}\right)}{2}$. By expectation, we should get $\tau_{s}\approx \frac{1}{\lambda }$, i.e., the estimated arrival rate is $\hat{\lambda }=\frac{1}{\tau_{s}}$.

Combining the two results above, the excess demand rate *μ*_*e*_ can now be estimated as ${\hat{\mu }}_{e}=\left(\hat{\lambda }+\hat{\mu }\right)-\hat{\lambda }=\frac{1}{\tau_{m}}-\frac{1}{\tau_{s}}$. However, it is possible that $\frac{1}{\tau_{m}}<\frac{1}{\tau_{s}}$. This happens when the inter-arrival intervals are very short, i.e., departure rate is relatively low compared with arrival rate. However, such low departure demand indicates there is not really any excess bike rental demand, or in other words the total demand can be reflected by the rentals observed. Finally, combining all of the above observations, the estimated excess demand rate is given by:

$$\begin{array}{c} {\hat{\mu }}_{e}=\max\left(\frac{1}{\tau_{m}}-\frac{1}{\tau_{s}},0\right) \end{array}$$ (1)

**Evaluation on synthetic data**. Since we do not have the ground truth for the excess demand in real data (i.e., people that attempted to rent a bike but the station was empty), we rely on simulations to evaluate whether Eq (1) is able to accurately estimate *μ*_*e*_. Our simulator begins with 0 available bikes at time *t* = 0 and ends at *t*_*end*_. The simulator operates as follows:

- **Time to next event**: We sample an exponential distribution with average $\frac{1}{\lambda +\mu }$, to generate a random interval *τ*_*r*_ that represents the time duration until the next event (either an arrival or a departure).
- **Event type**: We next have to *decide* the type of event happening. For this we sample a number *r*_*e*_ from a uniform distribution between 0 and 1. If $r_{e}<\frac{\lambda }{\lambda +\mu }$ we label the next event as an arrival, otherwise it is a departure. We also update the count of available bikes *a*.
- **Excess demand**: If *a* = 0, i.e., there are no available bikes, the next event cannot be a departure. Every time (when *a* = 0) the next event is simulated as a departure, we mark it as a failed bike departure. This will allow us to simulate the ground truth for the excess demand.

We simulate 1,000 time points (i.e., *t*_*end*_ = 1000 hours), while we use *μ* = 3 bikes/hour, λ = 1 bikes/hour. By setting *μ* > λ, we can create several situations where the bike rental demand cannot be fulfilled hence generating excess demand. Finally, we repeat the simulation 400 times.

In each simulation we collect the following information:

- The average *τ*_*s*_ of all the inter-arrival intervals.
- The average *τ*_*m*_ of all EDP lengths (i.e., *t*_2_ − *t*_1_ in Fig 4).
- We estimate the excess demand rate ${\hat{\mu }}_{e}$ using Eq (1).

**Fig 5:**
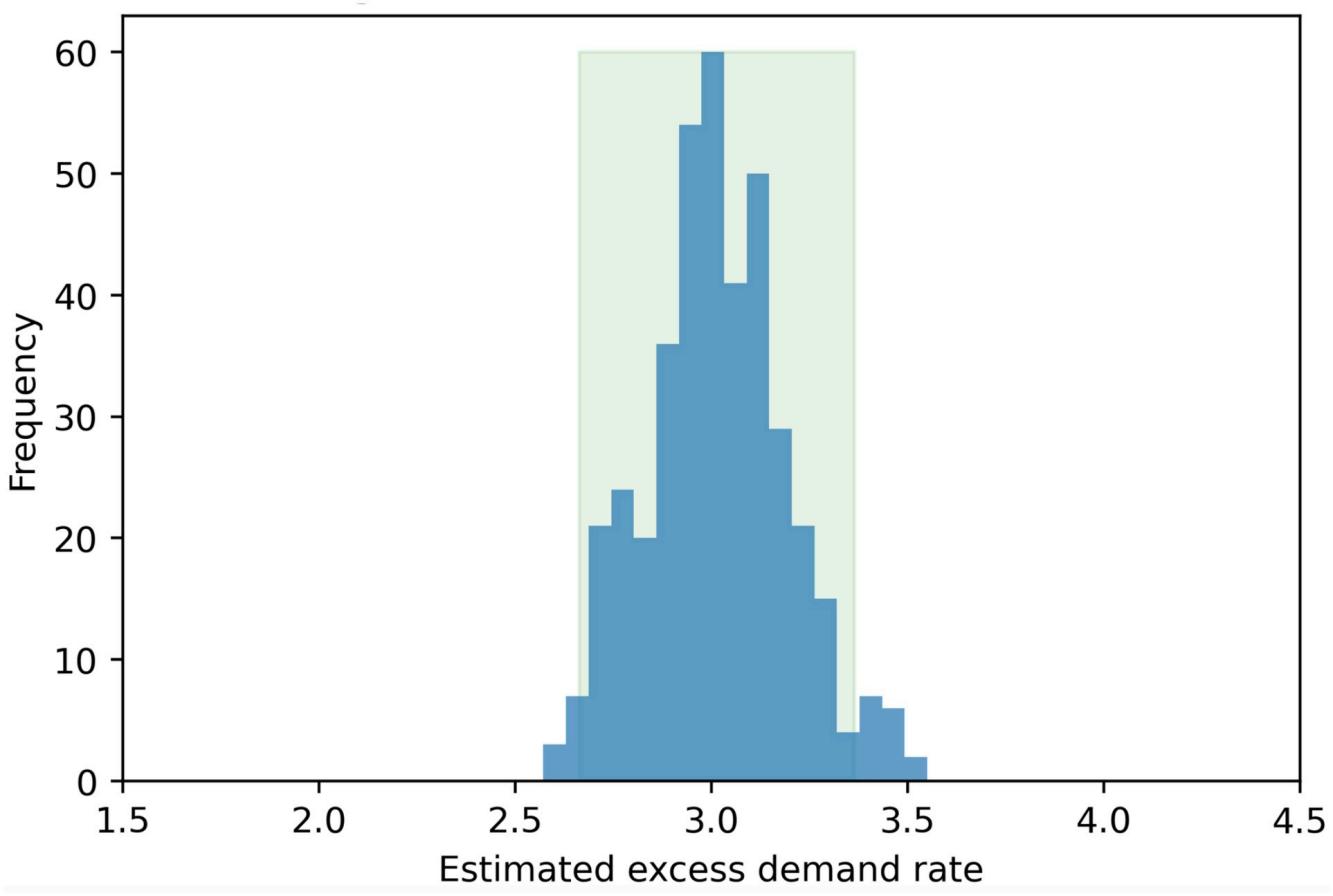
Histogram of estimated excess demand rate.

In our setting, since we assume that the demand is constant at 3 bikes/hour, the excess demand is also 3 bikes/hour. Simply put, even if we do not observe any departure for a prolonged period of time in our simulation when *a* = 0, there will be a constant demand of 3 bikes/hour during these intervals. Fig 5 depicts the distribution of ${\hat{\mu }}_{e}$ from each of our simulations. As we can see the distribution is centered around 3 bikes/hour, with an average of 3.014 bikes/hour (95% CI [2.66, 3.37]). Simply put, the proposed approach is able to estimate the true excess demand in our simulations, showcasing its appropriateness for the task at hand.

#### Excess demand in real data

Next we are interested in applying the aforementioned approach of excess demand estimation to data from a bike sharing operator. We use data from Divvy, the bike sharing system in Chicago, and in particular we collect:

- Historical bike trip records recorded on the system [36]. A bike trip record is a tuple including the following information: <start station ID, start station name, end station ID, end station name, start time stamp, end time stamp>.
- Historical bike station status data using the Chicago Data Portal API [37]. A record of station status is a tuple of the following form: <time stamp, station ID, station name, station coordinate, number of available bikes, number of free docks, number of docks occupied by bikes>. The status of each station is recorded every 10 minutes.
- Weather data from Openweathermap [38]. Each record is a tuple including the following information: <time stamp, temperature, humidity, pressure, descriptive weather conditions>.

**Fig 6:**
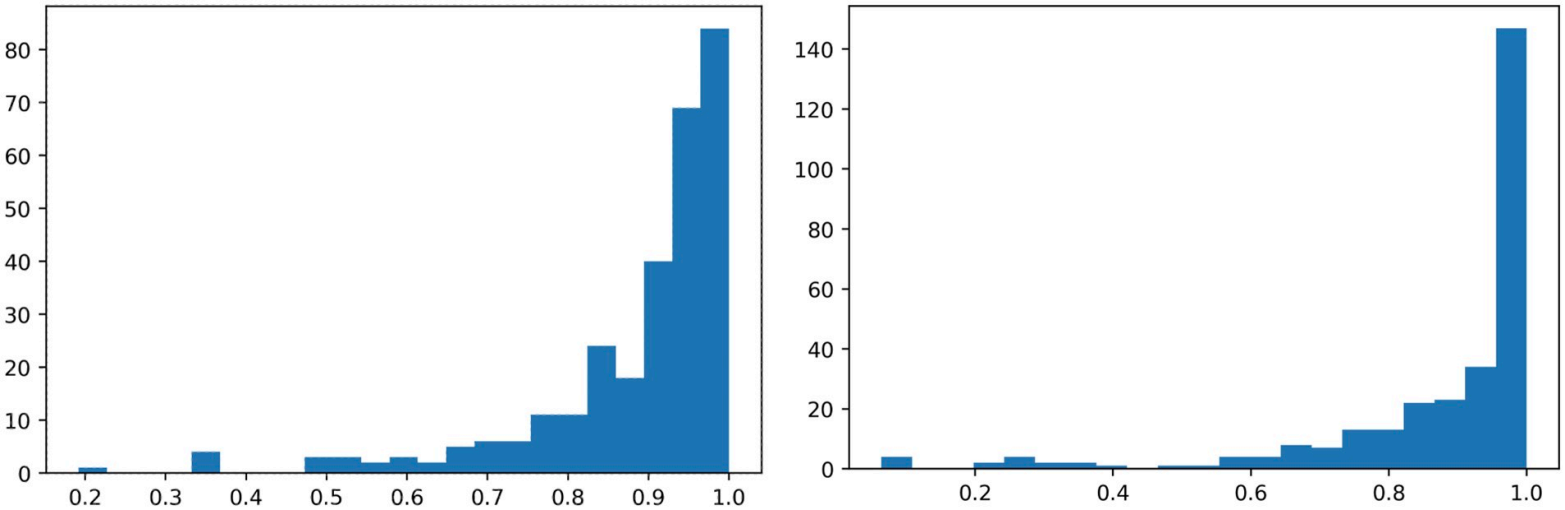
Average p-values from the K-S test for all stations for departures (left) and arrivals (right). The K-S test cannot reject the hypothesis that the observed data follow a Poisson distribution.

**Distribution of trips in Chicago’s Divvy**. Through our analysis above we have assumed that the trips’ departures and arrivals follow a Poisson distribution. We now statistically examine the validity of this assumption. More specifically, for a given station *j* and a given time period *t* (e.g., 9–9:30am), we first focus on the number of departure trips *n*_*j*,*t*_. By daily collecting observations for *n*_*j*,*t*_ during a given quarter (in order to avoid seasonality), we obtain a sequence {*n*_*j*,*t*_}. We calculate the average $\hat{n}$ of this sequence. We consequently repeatedly sample a Poisson distribution with mean $\hat{n}$ to generate *B* = 500 sequences of the same length as the observed one denoted as {*r*_*j*,*t*_}. We then compare the distribution of the observed departures {*n*_*j*,*t*_} and the Poisson sampled ones {*r*_*j*,*t*_} using two-sample K-S test [34]. Repeating this process for every station *j* we obtain the average p-value ${\hat{p}}_{j}$ for the null hypothesis that the observed sequence follows a Poisson distribution. Fig 6 (left) visualizes the distribution of these p-values for all the stations in the Divvy system. As we can see they are all larger than 0.2, which means that the test cannot reject the hypothesis that the observed data follow a Poisson distribution. We repeat the same process for the arrival events and Fig 6 (right) presents the results, where we can see that again we cannot reject the null hypothesis of the arrival data following a Poisson distribution.

These results verify that we cannot reject the hypothesis that the observed bike demand and supply in the Divvy system follow a Poisson distribution. However, we also make the assumption that the excess demands follow a Poisson distribution (possibly with a different rate). Given the sparsity of the excess demand data for each station and time period, the K-S test potentially fails to reject the null hypothesis due to reduced statistical power. However, it is a very reasonable assumption that the excess demand/supply will also be following the same distribution (albeit with different parameters) as the observed demand/supply.

**Estimating excess demand of bikes in Chicago’s Divvy**. Following the aforementioned approach of excess demand estimation, we can calculate the excess demand observed on the system. While the bike availability curves are just like the ones we simulated, there is one important difference. The excess demand rate in the real environment is not constant over time but it rather changes. For example, we expect the excess demand rate in the morning (rush hour) is higher than that in the late night. There are several factors that can lead to this temporal variation, ranging from people’s schedule (e.g., during rush hours the excess demand is expected to be higher) to weather conditions that change during the day. This temporal dependency does not allow us to use all *τ*_*f*_ intervals in the data to estimate a single, constant, excess demand. We will need to only use limited information, localized in time, to estimate the excess demand rate during a specific time interval.

In particular, we adjust the aforementioned approach in this section as follows. Here we still use Fig 4 to describe the adjusted approach. The EDP in the interval (*t*_1_, *t*_2_) is able to inform us about the excess demand occurring in the immediately preceding interval (*t*_*d*_, *t*_1_). We can use Eq (1) to calculate excess demand rate in this interval. However, *τ*_*f*_ = *t*_2_ − *t*_1_ is the only EDP length that we can use to calculate *τ*_*m*_ given the time-varying nature. Furthermore, we need to calculate the average inter-supply interval *τ*_*s*_, which again needs to be temporally localized due to its time varying nature. For the setting in Fig 4 we have arrival events at *t*_*a*_, *t*_*b*_ and *t*_1_. Thus, we use inter-arrival intervals, i.e., (*t*_*a*_, *t*_*b*_) and (*t*_*b*_, *t*_1_), to obtain $\tau_{s}=\frac{\left(t_{b}-t_{a}\right)+\left(t_{1}-t_{b}\right)}{2}$. Finally, we calculate ${\hat{\mu }}_{e}$ of interval (*t*_*d*_, *t*_1_) using Eq (1).

The single EDP length aforementioned may cause the calculated excess demand rate to be extreme. For instance, if the bike was rented almost immediately after it was returned, then the excess demand rate would be calculated practically as infinite. While we could eliminate such observations—since most probably correspond to users that return the bike and re-rent it immediately just for time-limit purposes imposed by the operator—it is not clear what is the time threshold as a good standard to eliminate such observations (i.e., such extremely short EDP lengths). To avoid having to choose an arbitrary cutoff, we make use of the Bayesian average [39]. The Bayesian average is a weighted average between (i) the estimate obtained from the sample we have for the quantity of interest, and (ii) a prior belief for this estimate. The weights are the sizes of the samples respectively (for the prior it can be a sample size that is considered *stable*). As with any Bayesian analysis, the prior can be purely subjective, or uninformative etc., but it can also be calculated by data. In our case, we can focus on a period of time around the time interval of interest and estimate the excess demand for the same periods over a week. If our measurement of the interval of interest was an extreme outlier, then the prior will shrink the final estimate. For example, let us assume that we want to calculate the excess demand rate at 9:30–10:00am on a given day, which is referred to as *μ*_930_. First, using Eq (1) we calculate the excess demand rates of 9:30–10:00am (interval of interest), and 9:00–9:30am, 10:00–10:30am (periods near interval of interest) of the given day. This will give us 3 observations and an observed average *μ*_*obs*_. Then using Eq (1) we calculate the excess demand rates of 9:00–9:30am, 9:30–10:00am, and 10:00–10:30am every day since 6 days before the given day. This will essentially give us 18 observations and an estimated prior average *μ*_*prior*_. Combining these with the Bayesian average we will get our final estimate for *μ*_930_ as:

$$\begin{array}{c} {\hat{\mu }}_{930}=\frac{3\cdot \mu_{obs}+18\cdot \mu_{prior}}{21} \end{array}$$ (2)

Of course, the choice of prior can be different, but the idea is that using this approach we can smooth extreme cases in a principled way. In the “S1 Text”, we further discuss how we processed instances that do not follow exactly the shape of EDP discussed here but appear infrequently in the data (e.g., when multiple bikes simultaneously arrive at a station as a result of rebalancing from the operator).

#### Estimating excess demand of docks in Divvy

Chicago bike sharing system does not allow for self-docking [11, 12]. Thus, if a bike is returned and the dock is full, there is no way to return it, leading to excess demand for the dock. To calculate the excess demand of docks, we can still use the method used to estimate the excess demand for bikes, but we need to make the following adjustments:

- The availability curve now represents dock availability (i.e., how many racks at the station are free), rather than bike availability (i.e., how many bikes are available at the station for renting).
- 0 dock availability means that each rack at the station is occupied by a bike.
- The EDP starts with a bike departure (from a full station) and quickly ends with a bike arrival. This allows us to capture how *quickly* the rack is being utilized again, thus, capturing, the excess demand for docks (which again is time-varying).
- *τ*_*f*_ still denotes EDP length (based on the definitions above), while *τ*_*m*_ still denotes average value of *τ*_*f*_.
- *τ*_*s*_ still denotes the average value of inter-supply intervals, but to reiterate, based on the definitions above, in this case a supply is a bike departure. So specifically, *τ*_*s*_ means the average value of inter-departure intervals.
- We use λ_*e*_ to denote excess demand rate of docks, which is formally defined in Eq (3):
  $$\begin{array}{c} {\hat{\lambda }}_{e}=\max\left(\frac{1}{\tau_{m}}-\frac{1}{\tau_{s}},0\right) \end{array}$$ (3)

**Fig 7:**
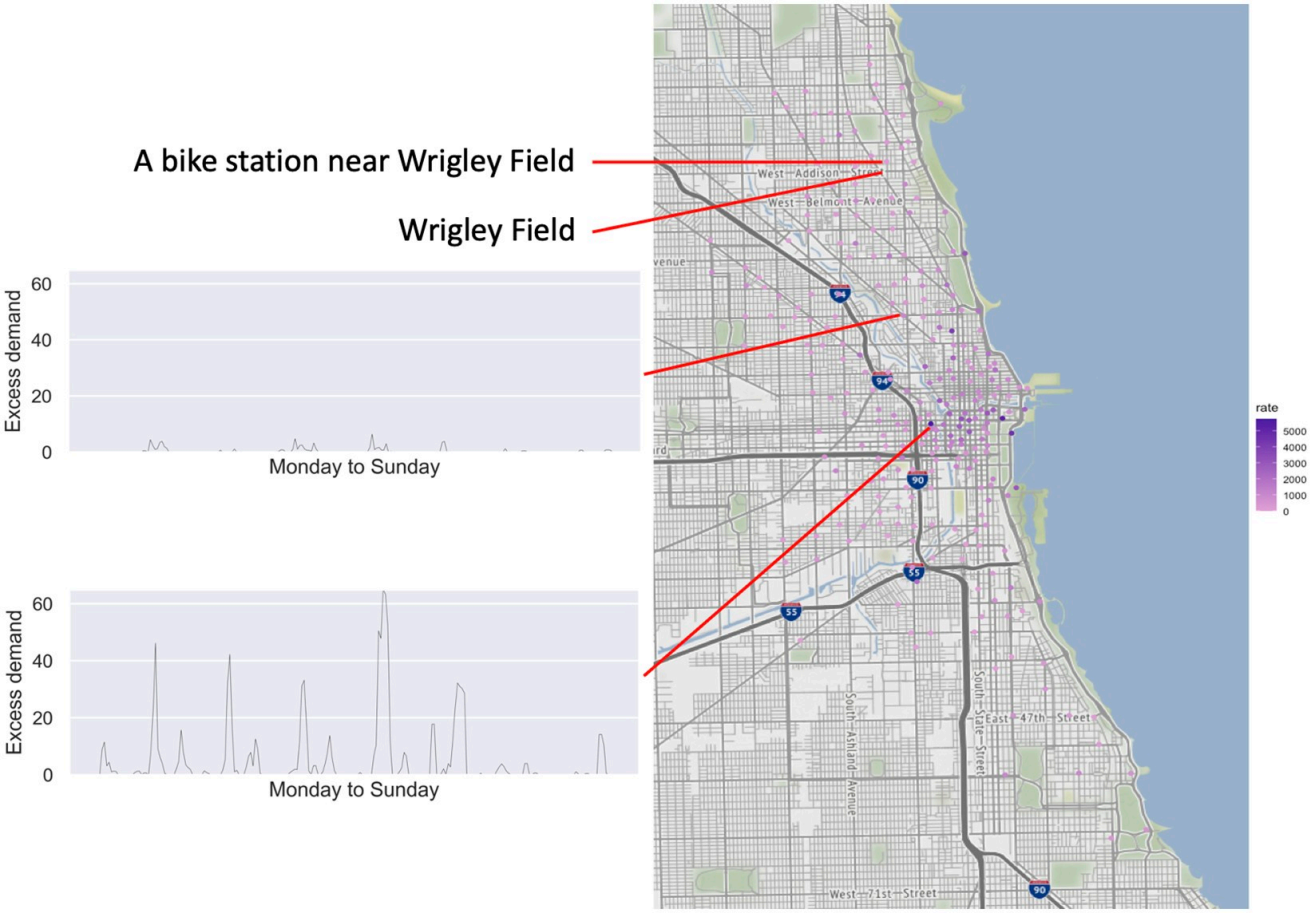
Cumulative bike excess demand rate for different stations. Reprinted from [40] under a CC BY license, with permission from OpenStreetMap, original copyright 2021.

**Fig 8:**
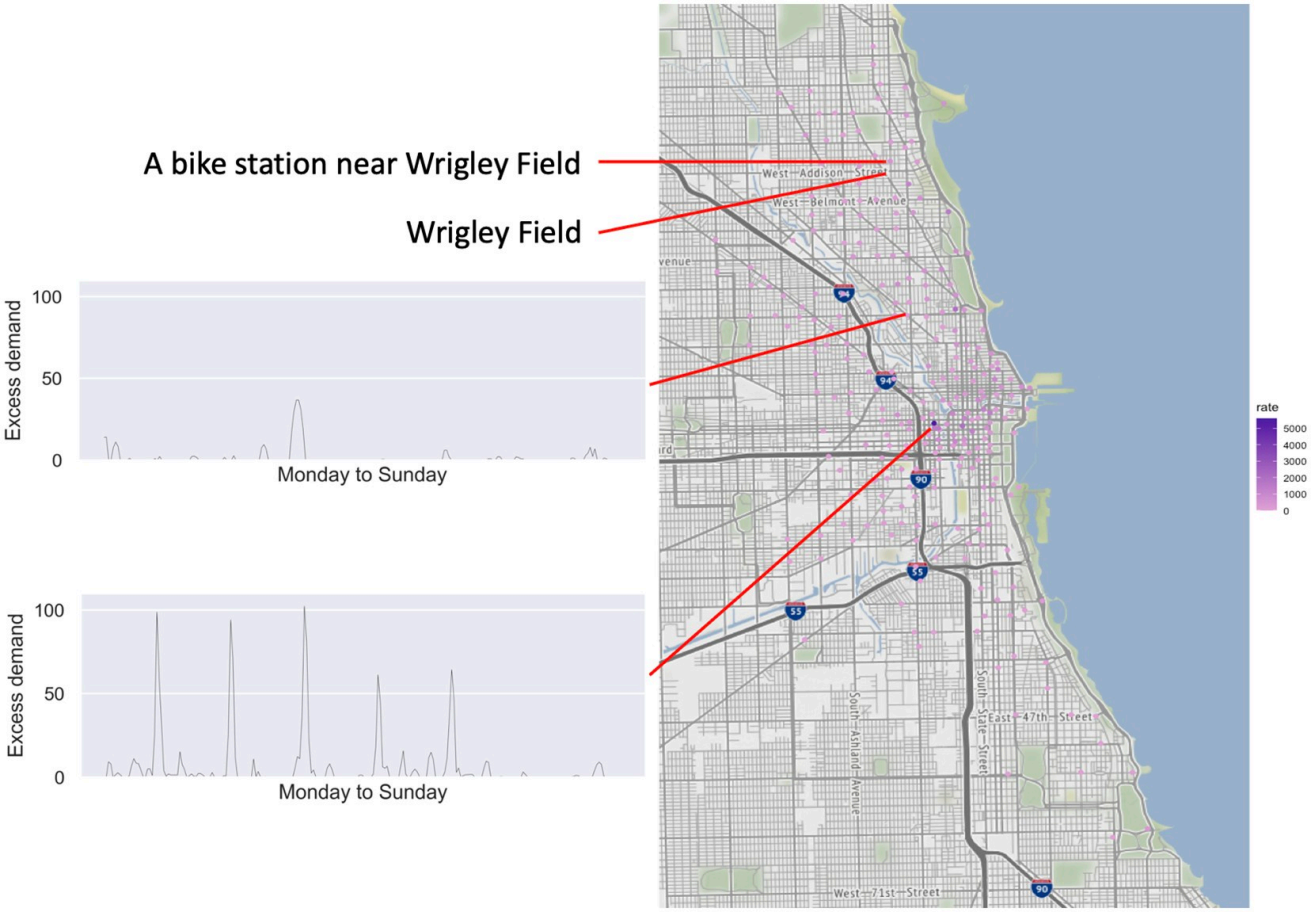
Cumulative dock excess demand rate for different stations. Reprinted from [40] under a CC BY license, with permission from OpenStreetMap, original copyright 2021.

**Excess demand in different stations**. As one might expect, the excess demand rates differ among different stations. The maps in Figs 7 and 8 illustrate the sum of the excess demand for each station for bikes and docks respectively. As we can see, stations closer to the downtown area have higher excess demand rate. We further illustrate in the inset figures the weekly patterns of the excess demand in 30-minute periods for two representative stations. As we can see these stations exhibit very different patterns in terms of levels of excess demands (both for bikes and docks). However, the relative spikes in each station appear to be similar to an extent. Furthermore, when focusing on a specific station, there seems to be a temporal shift between the excess demand for bikes and docks.

**Excess demand and sporting events**. In order to provide some context for the excess demand observed at the system, we examined the estimated excess demand near the Wrigley Field during game days. For example, at 1:20pm on July 8, 2018, there was a baseball game in Wrigley Field between the Cubs and the Reds [41]. There is a Divvy station only 130 meters away from Wrigley Field, which we have also marked in Fig 7. Based on our calculations, this station exhibited excess demand during particular time periods on that day. In particular, between 12:30pm and 2pm there was an average excess dock demand of more than 3 docks/30 minutes. This is possibly due to fans riding bikes to Wrigley Field, leading to non-empty docks. Furthermore, between 4pm and 5pm there was an average bike excess demand of 2.36 bikes/30 minutes, which is possibly due to several fans making their way out of the stadium as the game was coming to an end.

### Demand prediction models

The data processing described until now can facilitate a *post-hoc*, descriptive, analysis of the historical excess demand rates in a shared bike system. However, it is also important to explore the ability to perform predictions for the excess demand conditioned on various external variables. This can facilitate logistics operations, such as, rebalancing, fleet updates, etc. We define the following:

- Total bike demand volume *N*_*μ*_: Number of rented bikes in a 30-minute time interval of interest. This includes both bikes actually rented and bikes attempted to be rented but there was no availability.
- Total dock demand volume *N*_λ_: Number of returned bikes in a 30-minute time interval of interest. Again this includes both bikes actually returned, as well as, bikes attempted to be returned to a full station.
- Net total demand volume *Z* = *N*_*μ*_ − *N*_λ_: Difference between total bike demand volume and total dock demand volume in the same 30-minute time interval of interest.

In this section, we develop a predictive model for the net total demand volume at a station during a 30-minutes interval; i.e., build a predictive model for *Z* during a specific time interval. We choose *Z* as our dependent variable since it provides direct insights for the bike operator to decide the number of bikes to be rebalanced. Therefore, we need to estimate the bike and dock demand volumes during each 30-minute period in our data. However, it is important to note that these total demand volumes, include both the observed from the trip logs demand, as well as the excess demand that is not directly captured in these data. In particular, we perform the following steps for each 30-minute interval in our data:

- **Calculate observed demand volumes**: We obtain the number of observed departures, which is equal to the observed bike demand volume $N_{\mu_{o}}$ during the interval of interest, as well as, the number of observed arrivals, which is equal to the observed dock demand volume $N_{\lambda_{o}}$ for the same interval.
- **Calculate the excess demand rate**: As per the discussion in the previous section, we also identify EDPs from bike and dock availability to calculate bike and dock excess demand rates *μ*_*e*_ and λ_*e*_ respectively.
- **Convert rate to volume**: If a time duration with the existence of excess demand (i.e., a duration with 0 availability) is located inside our 30-minute interval of interest, we denote the length of that duration for bike, dock excess demand as $l_{\mu_{e}}$, $l_{\lambda_{e}}$, respectively. Then, we convert bike and dock excess demand rate to bike ($N_{\mu_{e}}$) and dock ($N_{\lambda_{e}}$) excess demand volume by multiplying with $l_{\mu_{e}}$, $l_{\lambda_{e}}$:
  $$\begin{array}{c} \begin{array}{c} N_{\mu_{e}}=\mu_{e}\times l_{\mu_{e}}\\ N_{\lambda_{e}}=\lambda_{e}\times l_{\lambda_{e}} \end{array} \end{array}$$ (4)

Using the above, we finally calculate *N*_*μ*_, *N*_λ_, *Z* as:

$$\begin{array}{c} \begin{array}{c} N_{\mu }=N_{\mu_{o}}+N_{\mu_{e}}\\ N_{\lambda }=N_{\lambda_{o}}+N_{\lambda_{e}}\\ Z=N_{\mu }-N_{\lambda } \end{array} \end{array}$$ (5)

Following the above process, we are able to obtain the net total demand volumes in the Divvy system for each 30-minute interval during the 2018 year.

To build our prediction model for the net total demand volume, we consider a set of variables that are expected to be correlated with the demand for bikes and docks. More specifically, we use the independent variables listed in Table 2.

**Table 2:** Independent variable list. The first three variables are numerical, and the remaining are categorical.

Name	Description
temperature	temperature (unit: Kelvins)
cloud percentage	percentage of clouds in the sky
wind speed	wind speed (unit: meter/sec)
day of a week	day index of a week: Mon—Sun
interval index	30-minute interval index of a day (e.g., 6:00—6:30, 6:30—7:00 etc.)
holiday indicator	binary indicator of whether the record falls in weekend or federal holidays (1) or not (0)
cloud indicator	binary indicator of the weather being “cloud” (1) or not (0)
rain indicator	binary indicator of the weather being “rain” (1) or not (0)
mist indicator	binary indicator of the weather being “mist” (1) or not (0)
snow indicator	binary indicator of the weather being “snow” (1) or not (0)
thunderstorm indicator	binary indicator of the weather being “thunderstorm” (1) or not (0)

Each data record used to build our model describes a 30-minute interval of observations. Given that the weather data are only available on the top of the hour, we interpolate them for the half hour interval. Having identified the covariates to use in our model, we start by exploring two generalized linear models, namely, Poisson regression and Skellam regression. With the first approach, we model the total demand volumes for the bike and dock demand independently, while with the second approach we model directly their difference, i.e., the net total demand *Z*. We also explore and evaluate the predictive performance of a feed forward neural network and XGBoost on the same set of features.

#### Poisson regression

To estimate *Z*, an intuitive approach would be to predict the total bike departures *N*_*μ*_ and bike arrivals *N*_λ_, and then calculate *Z* = *N*_*μ*_ − *N*_λ_. Bike departures and arrivals have been widely modeled as Poisson flows [19, 28–33], so a Poisson regression is an intuitive candidate model. A Poisson regression essentially models the expected value of the dependent variable through a linear combination of a set of independent variables **X** as:

$$\begin{array}{c} \lambda_{Y}=e^{\alpha +(b\cdot X)} \end{array}$$ (6)

The parameters *α* and **b** are obtained through maximum likelihood estimation. We can also estimate the distribution for the dependent variable *Y* as:

$$\begin{array}{c} p\left(Y=k|X,b,\alpha \right)=\frac{e^{k\cdot (\alpha +(b\cdot X))}}{k!}\cdot e^{-e^{\alpha +(b\cdot X)}} \end{array}$$ (7)

In our case, we have two processes that we need to model, namely the bike demand and the dock demand. Therefore, we learn two separate regression models using the covariates described above. For the rest of the paper, we will refer to this model as the “Two-Poisson regression” model.

#### Skellam regression

The Two-Poisson regression model assumes that the two processes—rentals and returns—are independent and hence, we can model them separately. However, this is not necessarily the case (The correlation between total bike demand volume *N*_*μ*_ and total dock demand volume *N*_λ_ of a station can be up to 0.885), and in these situations the estimations will be biased [42, 43]. However, we can directly model variable *Z* through a Skellam distribution since it represents the difference between two Poisson distributions [44]. In fact, if (*X*, *Y*) ∼ *BP*(λ_1_, λ_2_, λ_3_), where λ_3_ captures the covariance between *X* and *Y*, then their difference *Z* = *X* − *Y* follows the Skellam distribution:

$$\begin{array}{c} P\left(z\right)=e^{-(\lambda_{1}+\lambda_{2})}\cdot {\left(\frac{\lambda_{1}}{\lambda_{2}}\right)}^{z/2}\cdot I_{z}\left(2\sqrt{\lambda_{1}\lambda_{2}}\right) \end{array}$$ (8)

where *I*_*z*_(*x*) is the modified Bessel function. What we can observe is that the distribution does not depend on the covariance (λ_3_) of the two Poisson distributions [44].

Therefore we can model the net total demand *Z* through a Skellam regression. In particular:

$$\begin{array}{c} \begin{array}{c} Z∼Skellam(N_{\mu },N_{\lambda })\\ \ln\left(N_{\mu }\right)=b_{1}\cdot X\\ \ln\left(N_{\lambda }\right)=b_{2}\cdot X \end{array} \end{array}$$ (9)

where **X** denotes independent variables. **b**_1_ and **b**_2_ denote the coefficients to be learnt. We fit the model using Maximum Likelihood Estimation. Implementation source code can be found at https://github.com/xinliupitt/skellam_regression.

## Results

In this section we will present our evaluation results for predicting the net total demand. We will evaluate the predictive performance across two dimensions:

- Peak—vs—non-peak hour predictions
- Training based on observed—vs—total demand

Specifically, for the latter, we are interested in quantifying the predictive gains achieved by considering the excess bike and dock demand, and not only using recorded bike rentals and returns.

### Peak and non-peak hours

Typically “peak-hours” for a transportation system include weekdays morning (7am-9:30am) and evening commute (4pm-6:30pm). However, for a bike sharing system there is also seasonality, especially during the summer months [45]. Our data also support this seasonality. In particular, the net total demand during peak hours in the summer months is approximately 6 times higher as compared to that during non-peak hours of the year. For this reason, our results for peak hours below will be focused on the summer months. Different peak hours also have different patterns across seasons. Given the imbalance between the records for the peak hours per season and non-peak hours (peak hours per season cover a little less than 15% of the observations), a single model would be *overwhelmed* by the latter and will not able to identify the peak hour patterns in different seasons. Hence, we build separate models for different time periods. In particular, we learn a single model for non-peak hours, while we build two separate peak hour models (one for the morning and one for the evening peak hours). Predicting the net total demand for (particularly) the peak-hour periods is very important for the bike share system operator for various management operations, such as conduct an effective rebalancing. For learning each model, we split the data from all 300 stations and use 80% of the them to train the model, 10% as the validation set to optimize the regularization shrinking parameter, and the remaining 10% for out-of-sample evaluation. All models use L1 regularization, while we use the mean squared error (MSE) as our loss. The Skellam model training process follows the regression training setup in “S2 Text”.

**Baseline models**. We compare our proposed modeling (Skellam regression) with the following four baselines: (i) two independent Poisson models (Section “Poisson regression”), (ii) a feed forward neural network, (iii) XGBoost, (iv) constant prediction. They are referred to as “Two-Poisson”, “Neural”, “XGBoost”, “Constant”, respectively in Table 3. For the models except constant prediction, we apply L1 regularization and use the validation set to optimize the shrinking parameter. In particular, the “Two-Poisson” model follows the regression training setup in “S2 Text”. For the neural network we use 5 hidden layers, 32 units per layer and a batch size of 32 for training. For XGBoost, we set the number of estimators to 10,000. For the constant prediction, we use the average net total demand existing in the training set as our prediction for each out-of-sample record.

**Table 3:** MSE of different time periods.

Model type	Excess	All records	Excess	All records	Excess	All records
	(7–9:30)	(7–9:30)	(16–18:30)	(16–18:30)	(non-peak)	(non-peak)
Skellam	**36.2**	**6.4**	**36.4**	**10.3**	42.6	**2.7**
Two-Poisson	37.6	6.7	37.2	10.6	45.3	2.8
Neural	40.1	6.8	39.6	10.8	43.1	2.8
XGBoost	36.3	9.3	43.1	16.2	**40.2**	3.4
Constant	44.6	8.8	68.2	16.7	67.0	3.1

Table 3 presents the MSE on the test set for two peak hours periods (in the columns marked with “7-9:30”, “16-18:30”) as well as the non-peak hours (in the columns marked with “non-peak”). For each period, we present the MSE over all the records in the test set (in Table 3 columns marked with “All Records”). In the test set, there are some records with non-zero excess demand; that is, when calculating the ground truth *Z* of those records, either $N_{\mu_{e}}$ or $N_{\lambda_{e}}$ is non-zero. To understand better any gains existing in predictions, we also specifically present the MSE of those records (in Table 3 this corresponds to the columns marked with “Excess”). As aforementioned these instances are very important for the bike sharing system operator, since these are the situations where operations such as rebalancing are crucial. Note that the records with non-zero excess demand occupies 10% of the dataset for peak hours, and 2% for non-peak hours. For two peak-hour periods, as we can observe, the Skellam regression exhibits the lowest error among all the models examined. The benefits are even larger, in situations where the excess demand is non-zero. For non-peak hours, as we can see, Skellam exhibits only slight benefits over the two-Poisson model and the neural network. This could be attributed to the fact that during non-peak hours, there is an overall low demand for the bike sharing system, and hence, the two-Poisson and neural network models can capture this signal. Finally, XGBoost seems to perform slightly better than Skellam regression for records with non-zero excess demand. However, these records only occupy 2% of the dataset for non-peak hours (these could represent situations where there are special events—e.g., summer street fairs—that boost demand during non-peak hours).

**Fig 9:**
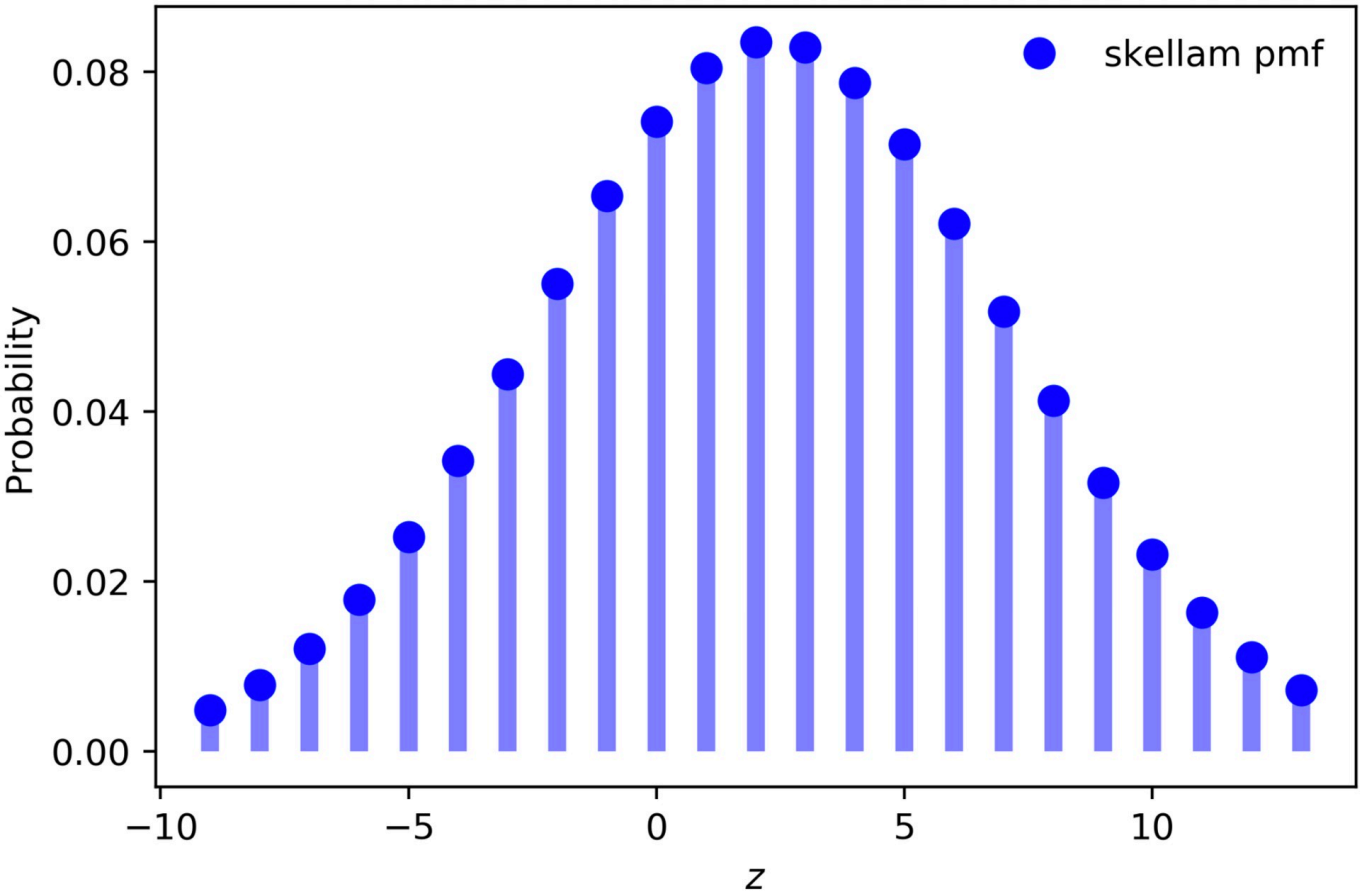
Skellam probability distribution with parameters ${\hat{N}}_{\mu }=12.67$, ${\hat{N}}_{\lambda }=10.29$. $\hat{Z}=2.38$.

Apart from its performance in terms of MSE, Skellam regression has two additional advantages over the alternative models considered. First, the Skellam regression as a generalized linear model is interpretable. This is particularly important from an operator’s perspective, since it can lead to actionable insights. For example, in a model built for a station during non-peak hours, for the independent variable “temperature” we obtain two coefficients: **b**_1,*temp*_ = 6.57 for bike demand and **b**_2,*temp*_ = 6.90 for dock demand (Eq (9)). These coefficients indicate that higher temperature is correlated with more people renting bikes for biking, i.e., higher bike demand. Since these riders need to return the bikes, the dock demand is also positively correlated with the temperature. Secondly, and most importantly, the Skellam regression model allows us to get a better estimation of the uncertainty of our prediction. In particular, we do not only get a single point estimate for the expected value of the net total demand, but rather its whole probability distribution. For example, let us assume that our predictions are ${\hat{N}}_{\mu }=12.67$ and ${\hat{N}}_{\lambda }=10.29$. This means that the net total demand is $\hat{Z}=2.38$. Recall, that ${\hat{N}}_{\mu }$ and ${\hat{N}}_{\lambda }$ are the two parameters of the Skellam distribution, and hence, we can plot the probability mass function for *Z* as presented in Fig 9. This distribution allows us to answer questions, such as *“what is the probability that there will be excess demand during a specific time period?”*. Questions are important for the system operators, providing them with a more holistic view of the system.

### Total and observed demand in training

For the results we presented above, we use the total demand *N*_*μ*_ and *N*_λ_ to calculate the dependent variable *Z* = *N*_*μ*_ − *N*_λ_. One of the motivations for our study is the fact that excess demand is not directly available in the trip/dock availability logs obtained from the bike system operator. Therefore, a lot of existing literature simply uses the observed demand for building predictive models. For these models, 0 trips from a station during a period is an indicator of 0 demand, even though as we have seen this may very well be an instance of actually high (excess) demand. However, what if even by simply using the observed demand to train our models, we can still get a good prediction for the net total demand. To examine this we build our model using only the observed demand when we train the model. We then evaluate the predictions on the test set and the results are presented in Table 4.

**Table 4:** MSE of different time periods under Skellam model.

Model type	Excess	All records	Excess	All records	Excess	All records
	(7–9:30)	(7–9:30)	(16–18:30)	(16–18:30)	(non-peak)	(non-peak)
Observed+Excess	36.2	6.4	36.4	10.3	42.6	2.7
Observed	47.5	10.0	52.2	11.9	45.8	2.9

As we can see, when training our models using the total demand (“Observed+Excess” in Table 4), the predictions have obvious performance gain (as expected). These gains are of course higher when making predictions for periods with excess demand, as one might have expected as well.

## Discussion and conclusions

In this paper, we introduce “excess demand” in bike sharing systems (e.g., how many customers attempted to rent a bike from an empty station). This type of demand is not directly recorded in bike trip logs. Key to our approach for estimating excess demand is identifying temporal segments in the bike availability data, that include changes in the availability from zero to non-zero. Through simulations, we verify the ability of our approach to estimate the excess demand present in the system. Consequently we apply our approach on data obtained from Chicago’s Divvy bike sharing system to estimate the excess demand present in Divvy system. To predict the net total demand (which includes the observed and excess demand), we learn a Skellam regression model through maximum likelihood estimation, which shows advantages over other alternative models, both in terms of predictive performance and interpretability. Moreover, our Skellam regression model, as a generalized linear model, allows us to get a better estimation of the uncertainty of our prediction, since we essentially obtain the whole probability distribution of our dependent variable.

Although we mainly use bike availability records to estimate the excess demand, additional data sources can potentially improve the excess demand estimation. For example, a customer of the bike sharing system may use the corresponding mobile application to explore the bike availability of stations near her location. This search itself is a signal of bike demand, and in the case where there are no available bikes nearby we can consider this to be part of the excess demand. Of course, a good understanding of the way the corresponding app operates is required, since for example if a local search is performed every time the app is turned on this is not necessarily an indicator of demand in the area. However, similar data are hard to be obtained as they are only available to the bike sharing operator. Furthermore, as implied from the results in our analysis of a specific baseball game at Wigley field, excess demand on one station might lead to spillover demand on nearby stations. When focusing on the whole bike sharing system this might lead to double counting—once as the excess demand of a station and once as the observed demand of another station. Our framework can be further improved by extending it so one can avoid this potential double counting of (total) demand.

Finally, while in this paper we focus on excess demand in docked bike sharing systems, excess demand also exists in dockless systems. In this setting we only need to consider the excess demand of renting bikes since bikes can be returned anywhere. However, this also means that there are no predefined stations. The lack of well-defined locations of bike demand provides additional challenges in analyzing these situations. The analysis should most probably focus on pre-defined areas within the city. However, identifying the spatial granularity needed is not a trivial task.

## Supporting information

S1 Text Special cases of excess demand. (PDF)

S2 Text Regression training setup. (PDF)
