# Supplementary material for: Excess demand prediction for bike sharing systems
Source: PLoS One. 2021 Jun 17;16(6):e0252894. doi: 10.1371/journal.pone.0252894 (PMC8211247; doi:10.1371/journal.pone.0252894)
Supplement: S1 Text — (PDF) [file pone.0252894.s001.pdf]

S1 Text. Special cases of excess demand. While the excess demand can be estimated as described in the main text for the vast majority of the instances, rebalancing from the operator can break down the calculations and a slightly different approach is needed.

For example, let us consider the situation in Fig 1. As we can see in this case, the availability at t_0 changes from 0 to k ($k \leqslant 4$ as observed in the real data of Divvy bike sharing system), potentially due to the bike sharing operator relocating/rebalancing k bikes to this station (of course, other reason are possible, such as, a group trip, but the treatment of the situation is the same regardless of the reason for causing it). Next the k bikes are consecutively consumed at times t_1, t_2, ...t_k respectively. This means that the demand rate is high, which leads to the supply of all the k bikes being consumed quickly before any new supply of bikes arrives. The EDP in this case is the curve between $[t_0, t_k]$, which to reiterate it is different from the *typical* EDP discussed in the main text. As we discussed in Section "Excess demand estimation", in this case the excess demand rate μ_e equals to the departure rate μ. The estimated departure rate $\hat{\mu}$ can be calculated by inverting the average of intervals between rentals; i.e., $[t_0,\, t_1]$, $[t_1,\, t_2]$, $[t_2,\, t_3]$, ..., $[t_{k-1},\, t_k]$. Then the average value of these intervals is $\dfrac{t_k - t_0}{k}$. Finally, the excess demand rate is estimated by inverting $\dfrac{t_k - t_0}{k}$, i.e., $\hat{\mu}_e = \dfrac{k}{t_k - t_0}$.

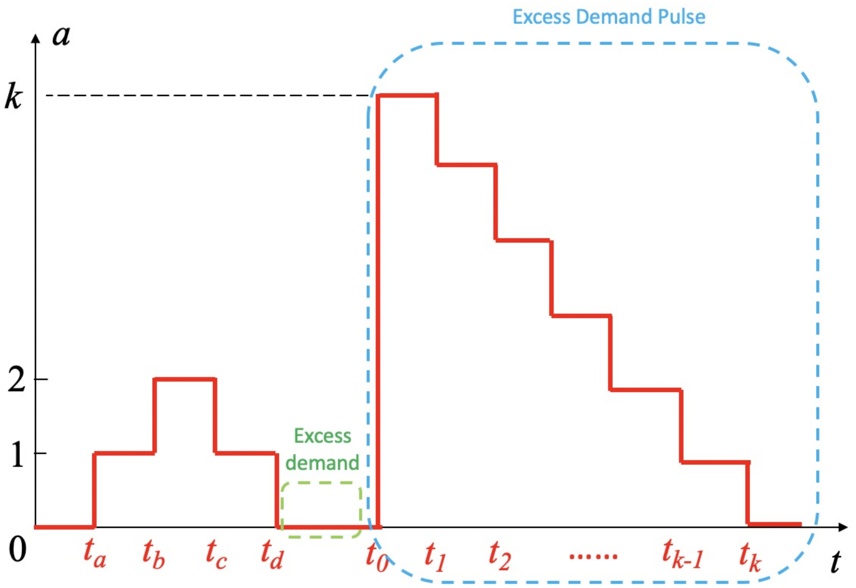

Fig 1. A segment of bike availability curve with a bulk of (potentially rebalanced bikes) arriving at t_0, all consumed by consecutive rentals.

The scenario shown by the bike availability curve in Fig 2 is a generalized case of Fig 1. The difference is that starting at t_0, the bikes are consecutively rented (at t_1, t_2, ...t_e respectively) up to the point when a supply arrives at t_g, where e is the total number of consumed bikes before t_g. In Fig 2, the EDP is the curve during $[t_0, t_e]$,

which is terminated by the supply arrival at t_g. The reason follows our explanation for Fig 3 in Section "Excess demand estimation". In particular, the supply arrival at t_g indicates that the bikes are not consumed *quickly* anymore and the ensuing rental would be recorded at the rental (observed) logs. Therefore, we calculate the excess demand using the departure records during $[t_0, t_e]$, when bikes are consumed *quickly*. Following the calculation method aforementioned the excess demand in this case is $\frac{e}{t_e - t_0}$.

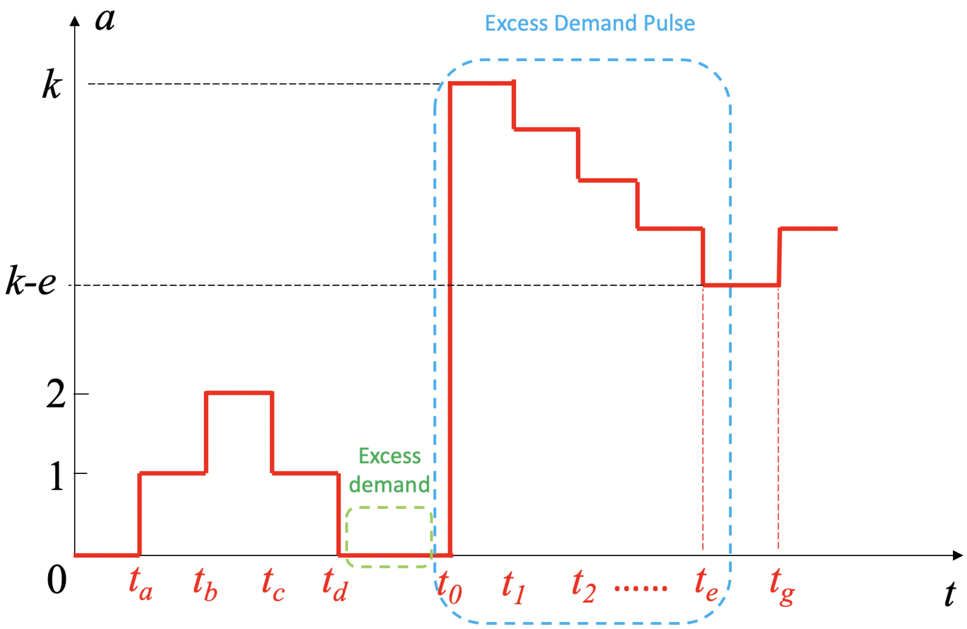

Fig 2. A segment of bike availability curve to describe the generalized case of excess demand with a bulk of bikes arriving at the dock at time t_0.
