# Supplementary material for: Excess demand prediction for bike sharing systems
Source: PLoS One. 2021 Jun 17;16(6):e0252894. doi: 10.1371/journal.pone.0252894 (PMC8211247; doi:10.1371/journal.pone.0252894)
Supplement: S2 Text — (PDF) [file pone.0252894.s002.pdf]

S2 Text. Regression training setup. For training the Skellam and the Two-Poisson regression models, we need the dependent variables to be integers, since the probability distribution for these models is discrete. However, from Eq (1)(3)(4), the values of our estimated excess demand volumes N_{μ_e}, N_{λ_e} can be non-integers, since they are obtained through the estimation of the excess demand rate. In order to be able to train the models we use sampling. In particular, the estimated excess demand volumes are the expected values of the Poisson processes for departures and arrivals. Therefore, we can sample two Poisson distributions with intensity N_{μ_e}, N_{λ_e} respectively, and obtain specific integer instances to update/replace N_{μ_e} and N_{λ_e}. This sampling process also incorporates some of the uncertainty around the excess demand volumes, captured by the whole probability distribution. Finally, we obtain integer instances for N_μ and N_λ as dependent variables of the Two-Poisson model, Z as dependent variable of the Skellam model.
